# Prognostic value of sarcopenia in patients treated by Radiochemotherapy for locally advanced oesophageal cancer

**DOI:** 10.1186/s13014-020-01545-z

**Published:** 2020-05-22

**Authors:** Romain Mallet, Romain Modzelewski, Justine Lequesne, Sorina Mihailescu, Pierre Decazes, Hugues Auvray, Ahmed Benyoucef, Fréderic Di Fiore, Pierre Vera, Bernard Dubray, Sébastien Thureau

**Affiliations:** 1 Department of Radiation Oncology and Medical Physics, Henri Becquerel Cancer Center and Rouen University Hospital, University of Rouen, CS11516 Rue d’Amiens, 76000 Rouen, France; 2 Department of Nuclear Medicine, Henri Becquerel Cancer Center and Rouen University Hospital & QuantIF–LITIS, University of Rouen, Rouen, France; 3 Clinical Research Department, Henri Becquerel Cancer Center, Rouen, France; 4 Department of Hepatogastroenterology, Rouen University Hospital & Department of Medical Oncology, Henri Becquerel Cancer Centre, University of Rouen, Rouen, France; 5 Department of Radiation Oncology and Medical Physics, Henri Becquerel Cancer Center and Rouen University Hospital & QuantIF–LITIS, University of Rouen, Rouen, France

**Keywords:** Sarcopenia, Chemotherapy, Radiotherapy, Oesophageal cancer, Overall survival, Morphological change

## Abstract

**Background:**

Sarcopenia is defined by a loss of skeletal muscle mass with or without loss of fat mass. Sarcopenia has been associated to reduced tolerance to treatment and worse prognosis in cancer patients, including patients undergoing surgery for limited oesophageal cancer. Concomitant chemo-radiotherapy is the standard treatment for locally-advanced tumour, not accessible to surgical resection. Using automated delineation of the skeletal muscle, we have investigated the prognostic value of sarcopenia in locally advanced oesophageal cancer (LAOC) patients treated by curative-intent chemo-radiotherapy.

**Methods:**

The clinical, nutritional, anthropometric, and functional-imaging (^18^FDG-PET/CT) data were collected in 97 patients treated between 2006 and 2012 in our institution. The skeletal muscle area was automatically delineated on cross-sectional CT images acquired at the 3rd. lumbar vertebra level and divided by the patient’s squared height (SML3/h^2^) to obtain the Skeletal Muscle Index (SMI). The primary endpoint was overall survival probability.

**Results:**

Seventy-six deaths were reported. The median survival time was 27 [95% Confidence Interval 23–40] months for the whole population. Univariate analyses (Cox Proportional Hazard Model) showed decreased survival probabilities in patients with reduced SMI, WHO > 0, Body Mass Index ≤21, and Nutritional Risk Index ≤97.5. Multivariate analyses showed that sarcopenia was the only significant prognostic factor (HR 2.32 [1.24–4.34], *p* = 0.008). Using Receiver Operating Characteristics curves, the Area Under the Curve (AUC) was 0.73 in males (*p* = 0.0002], the optimal threshold being 51.5 cm^2^/m^2^. In women, the AUC was 0.65 (*p* = 0.19).

**Conclusion:**

Sarcopenia is a powerful independent prognostic factor, associated with a rise of the overall mortality in patients treated exclusively by radiochemotherapy for a locally advanced oesophageal cancer. L3 CT images are easily gathered from ^18^FDG-PET/CT acquisitions.

## Introduction

Oesophageal cancer is the 19th most common cancer in the European Union (EU) [1], with 45 900 new cases diagnosed in 2012. It represents 1% of the total cancer in the EU [1]. The main histology is Oesophageal Squamous Cell Carcinoma (SCC), which represents 90% of oesophageal cancers worldwide, and Oesophageal Adenocarcinoma (OA), of which mortality rate has increased in several countries in the EU [2]. For patients with limited disease, the reference treatment is surgical resection [3]. For patients unable to undergo surgery, because of a locally advanced disease or a surgical contraindication, the reference treatment is chemoradiotherapy [3, 4], i.e. a combination of FOLFOX/ cisplatin-FU and 5-6 weeks radiotherapy (1.8-2 Gy/fraction, 5 days per week) [3]. Despite a decreasing rate of mortality (by 7% for EU men and 3% for EU women) [2], the prognosis remains poor with a median overall survival of 17.5-20.5 months [5]. Several prognostic factors have been studied in oesophageal cancer, especially nutritional factors [6–8]. Di Fiore et al showed that baseline nutritional status was predictive of response to treatment and survival in patients treated by definitive chemoradiotherapy for a locally advanced esophageal cancer (LAOC) [9].

Sarcopenia is defined by a loss of skeletal muscle mass with or without loss of fat mass [10]. Prado et al showed that sarcopenia at baseline, assessed by CT-scan of Skeletal Muscle Area (SMA) on the third lumbar vertebra, is a powerful prognostic factor for solid tumours of the respiratory and gastrointestinal tracts [11, 12].It is associated with reduced physical function [13], poor tolerance to anticancer therapy [14–17], and worse prognosis [18]. In operated oesophageal cancer patients, sarcopenia was associated to increased incidence of surgical complications and worse overall survival [19–22]. There are few studies on patients treated with chemoradiotherapy. A recent Japanese retrospective study shows the pejorative impact of pretherapeutic sarcopenia for patients with a LAOC [23]. We report a single-center retrospective analysis of 97 patients having received chemo-radiotherapy for LAOC. The presence of sarcopenia was assessed by automatically delineating the skeletal muscle area on CT slices at 3rd lumbar vertebra level retrieved a posteriori from staging ^18^FDG-PET/CT.

## Methods

### Patients and procedures

#### Objective(s)

A single-center study was performed at the Centre Henri Becquerel (Rouen, France). The population included all new LAOC patients referred to the Radiotherapy and Medical Physics department between October 5, 2005 and June 5, 2012. The inclusion criteria were: histologically confirmed oesophageal cancer, pre-therapeutic ^18^FDG-PET/CT with available images, and curative-intent chemo-radiotherapy. Surgical resection of residual tumour after chemo-radiotherapy was allowed.

The following baseline clinical data were collected: age, sex, WHO performance status, histological subtypes, TNM stage, and cancer location. The baseline nutritional parameters were: weight, size, Body Mass Index (BMI), weight loss history, serum albumin level, and the Buzby Nutritional Risk Index (NRI) [24]. We also collected treatment characteristics including the chemotherapy protocol and radiotherapy data (total dose, treatment duration). Finally, we collected imaging data from ^18^FDG-PET/CT, such as the Standardized Uptake Value Maximum (SUV _Max_), Total Volume of 40% of SUVmax segmentation (Tvol40), SUVmean, and Total Lesion Glycolysis (TLG).

**Fig. 1:**
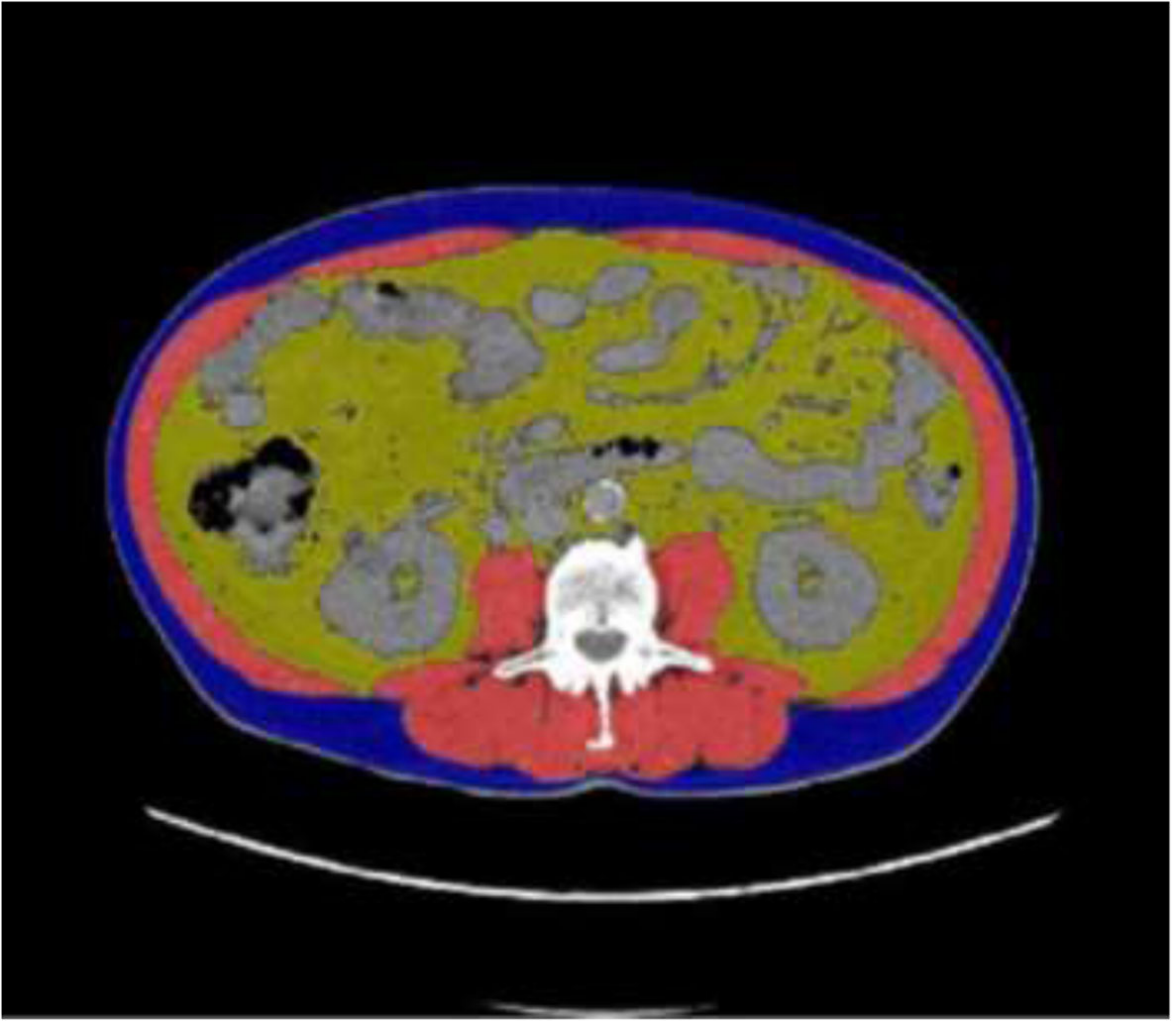
Example of SML (red), SCFML (blue) and VFM (yellow), measured on a cross-sectional images of the third lumbar vertebra

Sarcopenia was assessed by a homemade plugin running on our institutional Picture Archiving and Communication System (PACS, Telemis version 4.7, Telemis SA, Louvain la Neuve, Belgium). The skeletal muscles were automatically delineated by fixed thresholds (− 29 to + 150 Hounsfield Unit) [25] on two adjacent cross-sectional CT images acquired at the third lumbar vertebra (L3) and extracted from the staging ^18^FDG-PET/CT. The L3 skeletal muscles were the psoas, quadratus lumborum, paraspinal and abdominal wall muscles. The mean of the delineated surfaces on both images was defined as skeletal muscle L3 area (cm^2^). All delineations were visually checked and, if necessary, corrected by a single observer (RM). The Skeletal Muscle Index was calculated by dividing the skeletal muscle area by the squared height (SMI, cm^2^/m^2^) [25]. Sarcopenia was defined as SMI < 52.4cm^2^/m^2^ for men and < 38.5 cm^2^/m^2^ for women [26]. We also measured were the Mean Muscular Density (MMDL3, HU), the Visceral Fat Mass (VFML3, cm^2^) and the Subcutaneous Fat Mass (SCFML3, cm^2^) (Fig. 1). The cut-off values for SUVmax and Tvol40 by univariate analysis were based on Palie et al. [27].

The primary endpoint was overall survival, defined as the time from the start of the radiotherapy to death or last follow-up. Secondary endpoints were to define optimal cut-off values to predict the overall survival for SCFML3, VFML3 and mean muscular density.

All patients were irradiated at the Centre Henri Becquerel. Concomitant chemotherapy was delivered in the referring hospitals (CHU Rouen, CH Dieppe, CHI Elbeuf).

### Statistical analysis

Sarcopenic and non-sarcopenic patients were compared by Fisher’s exact and Pearson’s chi-square tests for categorical data, and by independent samples *t* tests or Mann-Whitney tests as appropriate for quantitative data. Survival probabilities were estimated by the Kaplan-Meier method and compared with the log-rank test. Univariate and multivariate analyses of variables associated to variations in survival were performed using the Cox model. In order to respect the TRIPOD criteria, the performance of the retained model was validated internally by a bootstrap method (1b statement). The Concordance index (C-index) was computed to assess discrimination between observed and predictive data. Predictive accuracy of death by SMI, SCFM, or VFM was assessed by Receiver Operating Characteristics ROC analysis. Optimal cut-off values were computed by maximizing predictive performance criteria (sensitivity Se, specificity Sp, Positive and Negative Predictive Values PPV and NPV). Two-sided tests and confidence intervals were reported at the 5% level of significance. All statistical analyses were performed using R software version 3.3.3 using the “survival” package for survival analysis, “boot” package for bootstrap analysis, and “ROCR” and “OptimalCutpoints” packages for ROC analysis.

#### Ethics and deontology

The study protocol RTEP3 has been approved by the Centre Henri Becquerel Cancer ethics committee (www.becquerel.fr).

## Results

From October 5, 2005 to June 5, 2012, 98 LAOC patients were referred for radiotherapy and 97 had available PET-CT images.

The overall characteristics of patients are shown in Table 1. A total of 194 CT L3 cross-sectional images were analysed. Fifty-four (56%) patients were classified as sarcopenic, and 43 (44%) as non-sarcopenic. Table 2 shows a predominance of male patients (81 men, 84%) and squamous cell carcinomas (77%). Sarcopenia was more prevalent in men than in women (49/81 (60%) vs 5/16 (31%), *p* = 0.05) and in squamous cell carcinomas (48/75 (64%) vs. 6/22 (27%), *p* = 0.04). The patients with sarcopenia had lower weights (mean 65 [15.2] kg. vs. 75 [12.3], *p* < 0.001), lower Body Mass Index BMI (22 [4.7] kg/m^2^ vs. 27 [4.2], *p* < 0.001), and larger weight loss (8% [0–32] vs. 5% [0–16], *p* = 0.02). The differences in albumin serum levels (38.42 [5.3] vs. 39.62 [39.6], *p* = 0.3) and Buzby NRI (97 [64–113] vs 101 [78–117], *p* = 0.08) were not statistically significant.

**Table 1:** Overall Characteristics of the population

	No. of patients, ***n*** = 97
**Clinical parameters**	
**Age**	63.61 (11.12)
**Sex**	
M	81 (83.5%)
F	16 (16.5%)
**Histological subtype**	
SCC	75 (77.3%)
OA	22 (22.7%)
**T**	
2	13 (14.4%)
3	74 (82.2%)
4	3 (3.3%)
**N**	
0	15 (15.6%)
1	75 (78.1%)
2	4 (4.2%)
3	2 (2.1%)
**M**	
0	80 (82.5%)
1	17 (17.5%)
**TNM Stage**	
I-II	25 (25.7%)
III-IV	72 (74.2%)
**Tumour location**	
Upper	20 (20.6%)
Mean	35 (36.1%)
Low	42 (43.3%)
**WHO Stage**	
0	46 (47.4%)
1	45 (46.4%)
> 1	6 (6.2%)
**Nutritional parameters**	
**NRI**	
> 97.5	41 (52.6%)
97.5–83.5	32 (41.0%)
< 83.5	5 (6.4%)
**Albumin levels (g/l)**	38.91 (5.14)
**Weight (kg)**	69.39 (14.84)
**Size (m)**	1.7 [1.45–1.85]
**BMI**	24.08 (5.00)
**Weight loss (%)**	7.00 [0.00–32]
**Anthropometric parameters**	
**SML3 (cm**^**2**^**)**	143.57 (29.15)
**VFML3 (cm**^**2**^**)**	109.33 [5.80, 418.29]
**SCFML3 (cm**^**2**^**)**	121.43 [4.20, 432.99]
**Mean Density (HU)**	30.59 (7.64)
**SMI**	49.59 (8.72)
^**18**^**FDG-PET/CT parameters**	
**TVol40**	13.50 [1.90, 140.40]
**SUVmean**	6.30 [1.90, 16.60]
**SUVmax**	11.50 [3.60, 27.90]
**TLG**	94.5 [6.9–1294]

Note: Qualitative variable are described by distribution and frequencies (%); Gaussian variables are described by mean and standard deviation; non-Gaussian variables are described by mean [range]

*Abbreviation*: *SCC* Squamous Cell Carcinoma, *OA* Oesophageal adenocarcinoma, *NRI* nutritional risk index, *BMI* Body Mass Index, *SML3* L3 Skeletal Muscular Mass, *VFML3* L3 Visceral Fat Mass, *SCFML3* L3 Subcutaneous Fat Mass, *SMI* Skeletal Muscle Index, *TNM* Tumour, Node, Metastasis, *WHO* World Health Organization, *TLG* Total Lesion Glycolysis

**Table 2:** Comparison between sarcopenic and non-sarcopenic patients

	Non Sarcopenic, ***n*** = 43 (%)	Sarcopenic, ***n*** = 54 (%)	p
**Clinical parameters**			
**Age**	61.84 (10.90)	65.06 (11.20)	0,158
**Sex**			0,061
M	32 (74.4%)	49 (90.7%)	
F	11 (25.6%)	5 (9.3%)	
**Histological subtype**			0,005
SCC	27 (62.8%)	48 (88.9%)	
OA	16 (37.2%)	6 (11.1%)	
**T**			0,177
1	0 (0%)	0 (0%)	
2	8 (20.0%)	5 (10.0%)	
3	32 (80.0%)	42 (84.0%)	
4	0 (0.0%)	3 (6.0%)	
**N**			0,567
0	9 (20.9%)	6 (11.3%)	
1	32 (74.4%)	43 (81.1%)	
2	1 (2.3%)	3 (5.7%)	
3	1 (2.3%)	1 (1.9%)	
**M**			0,066
0	39 (90.7%)	41 (75.9%)	
1	4 (9.3%)	13 (24.1%)	
**TNM Stage**			0,053
I-II	15 (34.9%)	10 (18.6%)	
III-IV	28 (65.1%)	44 (81.5%)	
**Tumour location**			0,151
Upper	6 (14.0%)	14 (25.9%)	
Mean	14 (32.6%)	21 (38.9%)	
Low	23 (53.5%)	19 (35.2%)	
**WHO Stage**			< 0.001
0	29 (67.4%)	17 (31.5%)	
1	11 (25.6%)	34 (63.0%)	
2	3 (7.0%)	2 (3.7%)	
3	0 (0.0%)	1 (1.9%)	
**Nutritional parameters**			
**NRI**			0,167
> 97.5	21 (65.6%)	20 (43.5%)	
97.5–83.5	10 (31.2%)	22 (47.8%)	
< 83.5	1 (3.1%)	4 (8.7%)	
**Albumin levels (g/l)**	39.62 (4.91)	38.42 (5.28)	0,301
**Weight (kg)**	75.23 (12.29)	64.74 (15.15)	< 0.001
**Size (m)**	1.70 [1.52, 1.85]	1.71 [1.45, 1.81]	0,112
**BMI**	26.52 (4.24)	22.14 (4.73)	< 0.001
**Weight loss (%)**	5.00 [0.00, 16.00]	8.00 [0.00, 32.00]	0,018
**Anthropometric parameters**			
**SML3 (cm**^**2**^**)**	158.78 (31.28)	131.45 (20.64)	< 0.001
**VFML3 (cm**^**2**^**)**	139.85 [10.70, 418.29]	82.06 [5.80, 415.74]	0,005
**SCFML3 (cm**^**2**^**)**	145.89 [55.13, 432.99]	81.94 [4.20, 419.88]	< 0.001
**Mean Density (HU)**	31.33 (7.40)	30.00 (7.85)	0,398
**SMI**	55.47 (8.12)	44.91 (5.93)	< 0.001
^**18**^**FDG-PET/CT parameters**			
**SUVmax**	11.20 [4.90, 26.40]	12.15 [3.60, 27.90]	0,17
**TVol40**	12.60 [2.00, 70.00]	17.85 [1.90, 140.40]	0,111
**SUVmean**	6.00 [1.90, 16.60]	7.10 [2.90, 16.50]	0,112
**TLG**	69.20 [7.40, 482.50]	121.50 [6.90, 1294.00]	0,044

Note: Qualitative variable are described by distribution and frequencies (%); Gaussian variables are described by mean and standard deviation; non-Gaussian variables are described by mean [range]

*Abbreviation*: *SCC* Squamous Cell Carcinoma, *OA* Oesophageal adenocarcinoma, *NRI* nutritional risk index, *BMI* Body Mass Index, *SML3* L3 Skeletal Muscular Mass, *VFML3* L3 Visceral Fat Mass, *SCFML3* L3 Subcutaneous Fat Mass, *SMI* Skeletal Muscle Index, *TNM* Tumour, Node, Metastasis, *WHO* World Health Organization, *TLG* Total Lesion Glycolysis

**Fig. 2:**
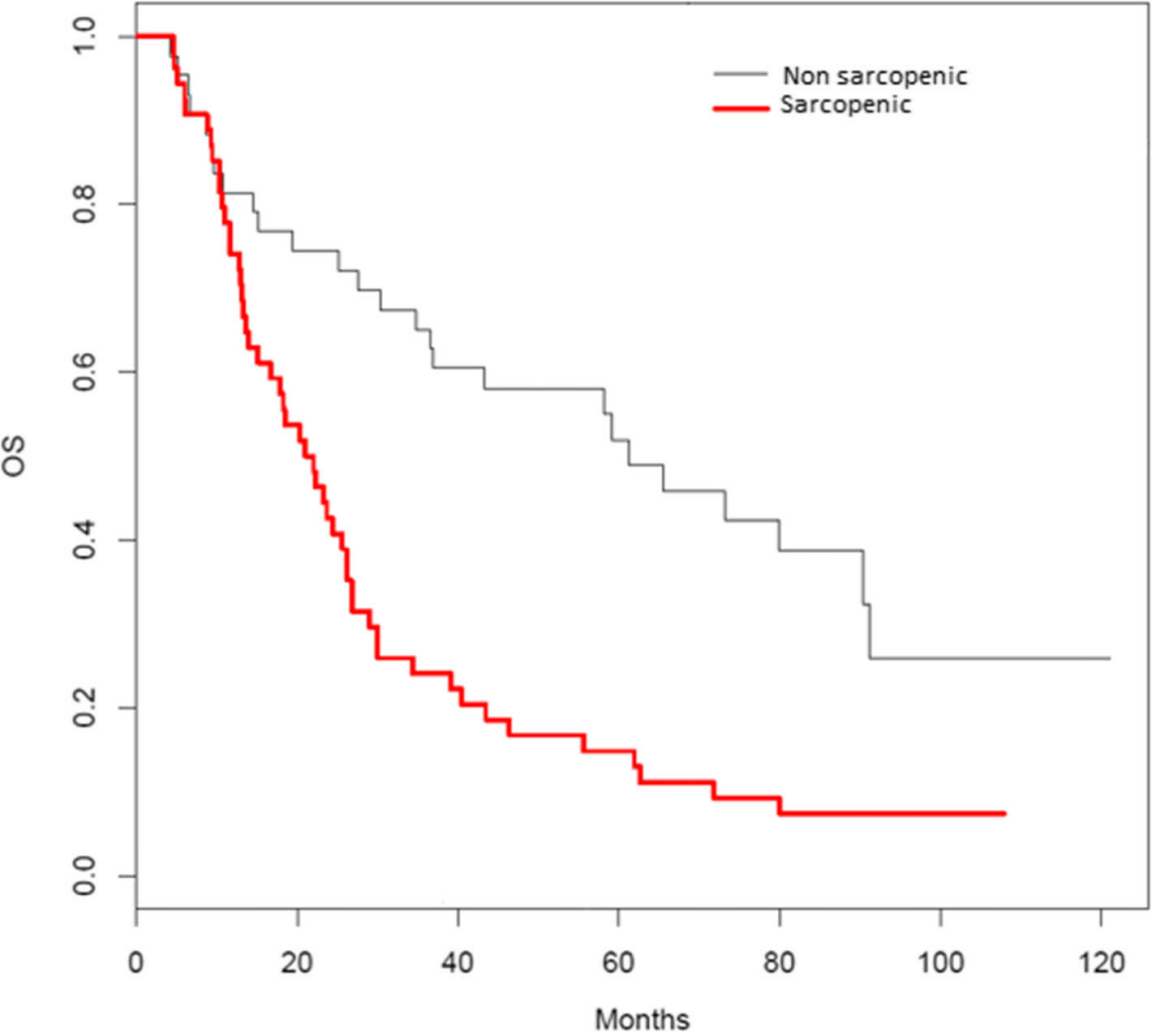
Overall survival of sarcopenic and non-sarcopenic patients

There were 76 deaths out of 97 patients, 50/54 (93%) in the sarcopenic population versus 26/43 (61%) in the non-sarcopenic population. The overall median survival was 27 (4–121) months, 22 months in the sarcopenic patients and 61 months in the non-sarcopenic patients (Fig. 2). There were 17 patients with metastasis (17.5%), of whom 4 were non-sarcopenic patients (23.5%) and 13 were sarcopenic (76.5%) (*p* = 0.06).

Univariate analysis (Table 3) shows an increased risk of death in patients with sarcopenia (Hazard Ratio HR 2.61 [95% Confidence Interval 1.61–4.24], *p* < 0.001), BMI < 21 (1.67 [1.03–2.73], *p* = 0.037), and NRI < 97.5 (1.71 [1.04–2.81], *p* = 0.033). The risk of death was lower in WHO = 0 patients (0.63 [0.4–0.99], *p* = 0.04). A multivariate analysis including these factors (Table 4) showed that sarcopenia was the only significant prognostic factor (HR 2.32 [1.24–4.34], *p* = 0.008). The model was found to have acceptable concordance during internal validation with C-index of 0.64 [0.56–0.72] based on 1000 bootstrap replicates.

**Table 3:** Hazard ratio and 95%CI for proportional hazard models assessing the effect of variables associated with survival (univariate analysis)

	Coefficient (SE)	Hazard ratio (95%CI)	p
BMI < =21	0.51 (0.25)	1.67 (1.03–2.73)	0.037
NRI < =97.5	0.54 (0.25)	1.71 (1.04–2.81)	0.033
WHO Score*	− 0.47 (0.23)	0.63 (0.4–0.99)	0.044
Sarcopenia****	0.96 (0.24)	2.61 (1.61–4.24)	< 0.001
Weight loss<=5%	−0.35 (0.24)	0.71 (0.44–1.13)	0.142
Weight loss<=10%	−0.13 (0.26)	0.88 (0.53–1.45)	0.604
Albumin levels<=35(g/l)	−0.23 (0.31)	0.79 (0.43–1.45)	0.449
BMI < =18	0.61 (0.33)	1.84 (0.96–3.53)	0.061
Masculine Sex	0.53 (0.34)	1.7 (0.87–3.3)	0.116
Age < =65	−0.3 (0.23)	0.74 (0.47–1.16)	0.186
Histology ***	0.32 (0.29)	1.38 (0.77–2.47)	0.277
SUV max<=12.3	−0.33 (0.23)	0.72 (0.46–1.13)	0.154
TVol40 < =9.9	−0.44 (0.28)	0.64 (0.37–1.12)	0.116
Muscular Mean Density < =41 (for BMI < 25)	0.02 (0.43)	1.02 (0.43–2.41)	0.957
Muscular Mean Density < =33 (for BMI > 25)	1.11 (0.74)	3.02 (0.71–12.82)	0.116
Metastasis **	−0.33 (0.33)	0.72 (0.38–1.37)	0.315

*Versus patients with WHO score > 0. ** Versus patient without metastasis. ***SCC versus OA. **** Defined as SMI < 38.3 cm^2^/m^2^ for women and < 52.4 cm^2^/m^2^ for men

**Table 4:** Hazard ratio and 95% CI for proportional hazard models assessing the effect of variables associated with survival (multivariate analysis)

	Coefficient (SE)	Hazard ratio (95%CI)	p
BMI < =21	0.08 (0.3)	1.09 (0.6–1.96)	0.78
NRI < =97.5	0.34 (0.28)	1.4 (0.81–2.42)	0.225
WHO Score*	−0.18 (0.28)	0.83 (0.48–1.44)	0.504
Sarcopenia****	0.84 (0.32)	2.32 (1.25–4.34)	0.008

*Versus patients with WHO score > 0. **** Defined as SMI < 38.3 cm^2^/m^2^ for women and < 52.4 cm^2^/m^2^ for men

**Fig. 3:**
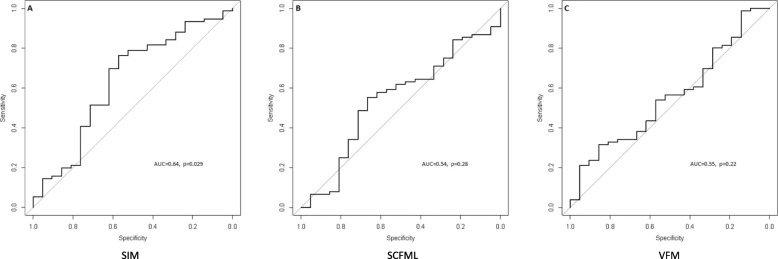
ROC curves for SMI (**a**), SCFML (**b**) and VFM (**c**)

ROC curve analyses are shown on Fig. 3. The Area Under the Curve (AUC) was 0.64 (*p* = 0.03) for SMI over the whole study population (Fig. 3 (a)). Of note, according to various criteria used to optimize sensibility and specificity, optimal cut-points varied from 50.55 (Se = 63, Sp = 62, PPV = 86, NPV = 32) to 51.98 cm^2^/m^2^ (Se = 70, Sp = 62, PPV = 87, NPV = 36) for the whole sample, and from 53.63 (Se = 74, Sp = 73, PPV = 92, NPV = 39) to 53.84cm^2^/m^2^ (Se = 76, Sp = 73, PPV = 93, NPV = 41) for men. The AUC for SMI over the 16 women included in the study was 0.4 (*p* = 0.28) so that optimal cut-point could not be investigated. Similarly, the AUCs for VFML3 and SCFML3 (Fig. 3 (b) and (c)) were 0.54 (*p* = 0.28) and 0.55 (*p* = 0.22), respectively.

## Discussion

The present study demonstrates that the presence of sarcopenia is strongly associated to an increased risk of death in 97 patients having received curative-intent chemo-radiotherapy for LAOC. We have confirmed the observations made in operated oesophageal carcinoma patients [11, 12] and a previous study on chemoradiotherapy [23]. The prevalence of sarcopenia (44%) was consistent with the literature (26–57%) [11, 17–22],There was a difference for metastasis status between sarcopenic and non sarcopenic patients, although not significant. It could be a bias for survival comparison. However, of the 17 patients, there were 15 patients staged as M1 because of a non-regional positive lymph node involvement on PET-CT and 2 patients with a single pulmonary metastasis (one in each group). All these were considered accessible to a loco-regional treatment in a multidisciplinary team meeting, and we chose to include them as a reflection of the current medical practice.

The usual nutritional features serum albumin level, BMI, and weight loss were not significantly associated with survival on multivariate analysis, which may be explained by limited statistical power in our study. Indeed, BMI < 18 appeared not to be statistically associated with survival (*p* = 0.061) with an HR of 1.84 [0.96–3.53]. Moreover, trends concerning serum albumin levels and weight loss are in agreement with well-known results in the literature.

We performed an internal validation on our sample, in order to confirm the reliability of the model. Since the sample size was limited, we chose to use a boostrapping method. Internal validation showed an acceptable degree of reliability of the model, by using a boostrap method as recommended by TRIPOD statement for prediction development when dealing with small samples. External validation could not have however been performed in our study. The proposed statistical model thus deserves to be validated in other cohorts of patients.

The relation between sarcopenia and increased mortality is not fully understood. Increased susceptibility to nosocomial infection [28], baseline systemic inflammation associated to higher rates of metastasis and progression [29], and variations of chemotherapy volume distribution with sarcopenia [30, 31] are possible explanations. For example, the presence of sarcopenia was associated to higher 5-FU-induced toxicities [14]. A limitation of our study is that we could not collect data about toxicity. These data should be analysed on a prospective clinical trial which is to be confirmed. Sarcopenia can also be considered as reflect of the clinical state of the patient.

To conclude, sarcopenia assessed by CT sequence on PET-CT at baseline is an independent and robust prognostic factor of overall survival in patients with LAOC treated exclusively by radiochemotherapy, more prognostic than WHO score, BMI, albumin levels and weight loss. These easily gathered imaging features can identify an at-risk population who need a specific therapy. Despite this, evaluation of sarcopenia is not currently used because of the necessity of manual segmentation. Automatic segmentation software is necessary to expand its use.

## Data Availability

The data are available at Henri Becquerel Center, Unity of Clinic Research.

## Abbreviations

SMI: Skeletal Muscle Index
AUC: Area Under the Curve
EU: European Union
SCC: Squamous Cell Carcinoma
OA: Oesophageal Adenocarcinoma
LAOC: Locally advanced esophageal cancer
SMA: Skeletal Muscle Area
BMI: Body Mass Index (BMI)
NRI: Nutritional Risk Index
SUV max: Standardized Uptake Value Maximum
Tvol40: Total Volume of 40% of SUVmax segmentation
TLG: Total Lesion Glycolysis
L3: Third lumbar vertebra
MMDL3: Mean Muscular Density
VFML3: Visceral Fat Mass
SCFML3: Subcutaneous Fat Mass
BMI: Body Mass Index BMI
HR: Hazard Ratio

## References

[1] Ferlay J, Steliarova-Foucher E, Lortet-Tieulent J, Rosso S, Coebergh JWW, Comber H (2013). Cancer incidence and mortality patterns in Europe: estimates for 40 countries in 2012. Eur J Cancer.

[2] Castro C, Bosetti C, Malvezzi M, Bertuccio P, Levi F, Negri E (2014). Patterns and trends in esophageal cancer mortality and incidence in Europe (1980–2011) and predictions to 2015. Ann Oncol.

[3] Lordick F, Mariette C, Haustermans K, Obermannová R, Arnold D (2016). Oesophageal cancer: ESMO Clinical Practice Guidelines for diagnosis, treatment and follow-up†. Ann Oncol.

[4] Créhange G, Huguet F, Quero L, N’Guyen TV, Mirabel X, Lacornerie T (2016). Radiothérapie des cancers de l’œsophage, du cardia et de l’estomac. Cancer/Radiothérapie..

[5] Conroy T, Galais M-P, Raoul J-L, Bouché O, Gourgou-Bourgade S, Douillard J-Y (2014). Definitive chemoradiotherapy with FOLFOX versus fluorouracil and cisplatin in patients with oesophageal cancer (PRODIGE5/ACCORD17): final results of a randomised, phase 2/3 trial. Lancet Oncol.

[6] Dewys WD, Begg C, Lavin PT, Band PR, Bennett JM, Bertino JR (1980). Prognostic effect of weight loss prior to chemotherapy in cancer patients. Eastern cooperative oncology group. Am J Med.

[7] Andreyev HJ, Norman AR, Oates J, Cunningham D (1998). Why do patients with weight loss have a worse outcome when undergoing chemotherapy for gastrointestinal malignancies?. Eur J Cancer.

[8] Cox S, Powell C, Carter B, Hurt C, Mukherjee S, Crosby TDL (2016). Role of nutritional status and intervention in oesophageal cancer treated with definitive chemoradiotherapy: outcomes from SCOPE1. Br J Cancer.

[9] Di Fiore F, Lecleire S, Pop D, Rigal O, Hamidou H, Paillot B (2007). Baseline nutritional status is predictive of response to treatment and survival in patients treated by definitive Chemoradiotherapy for a locally advanced esophageal Cancer. Am J Gastroenterol.

[10] Fearon K, Strasser F, Anker SD, Bosaeus I, Bruera E, Fainsinger RL (2011). Definition and classification of cancer cachexia: an international consensus. Lancet Oncol.

[11] Prado CMM, Lieffers JR, McCargar LJ, Reiman T, Sawyer MB, Martin L (2008). Prevalence and clinical implications of sarcopenic obesity in patients with solid tumours of the respiratory and gastrointestinal tracts: a population-based study. Lancet Oncol.

[12] Martin L, Birdsell L, MacDonald N, Reiman T, Clandinin MT, McCargar LJ (2013). Cancer Cachexia in the age of obesity: skeletal muscle depletion is a powerful prognostic factor, independent of body mass index. J Clin Oncol.

[13] Baumgartner RN, Wayne SJ, Waters DL, Janssen I, Gallagher D, Morley JE (2004). Sarcopenic obesity predicts instrumental activities of daily living disability in the elderly. Obes Res.

[14] Prado CMM, Baracos VE, McCargar LJ, Mourtzakis M, Mulder KE, Reiman T (2007). Body composition as an independent determinant of 5-fluorouracil-based chemotherapy toxicity. Clin Cancer Res.

[15] Antoun S, Baracos VE, Birdsell L, Escudier B, Sawyer MB (2010). Low body mass index and sarcopenia associated with dose-limiting toxicity of sorafenib in patients with renal cell carcinoma. Ann Oncol.

[16] Prado CMM, Baracos VE, McCargar LJ, Reiman T, Mourtzakis M, Tonkin K (2009). Sarcopenia as a determinant of chemotherapy toxicity and time to tumor progression in metastatic breast Cancer patients receiving Capecitabine treatment. Clin Cancer Res.

[17] Yip C, Goh V, Davies A, Gossage J, Mitchell-Hay R, Hynes O (2014). Assessment of sarcopenia and changes in body composition after neoadjuvant chemotherapy and associations with clinical outcomes in oesophageal cancer. Eur Radiol.

[18] Makiura D, Ono R, Inoue J, Kashiwa M, Oshikiri T, Nakamura T (2016). Preoperative sarcopenia is a predictor of postoperative pulmonary complications in esophageal cancer following esophagectomy: a retrospective cohort study. J Geriatric Oncol.

[19] Nishigori T, Okabe H, Tanaka E, Tsunoda S, Hisamori S, Sakai Y (2016). Sarcopenia as a predictor of pulmonary complications after esophagectomy for thoracic esophageal cancer: sarcopenia and esophageal Cancer. J Surg Oncol.

[20] Elliott Jessie A., Doyle Suzanne L., Murphy Conor F., King Sinead, Guinan Emer M., Beddy Peter, Ravi Narayanasamy, Reynolds John V. (2017). Sarcopenia. Annals of Surgery.

[21] Tamandl D, Paireder M, Asari R, Baltzer PA, Schoppmann SF, Ba-Ssalamah A (2016). Markers of sarcopenia quantified by computed tomography predict adverse long-term outcome in patients with resected oesophageal or gastro-oesophageal junction cancer. Eur Radiol.

[22] Paireder M, Asari R, Kristo I, Rieder E, Tamandl D, Ba-Ssalamah A (2017). Impact of sarcopenia on outcome in patients with esophageal resection following neoadjuvant chemotherapy for esophageal cancer. Eur J Surg Oncol (EJSO).

[23] Onishi Sachiyo, Tajika Masahiro, Tanaka Tsutomu, Hirayama Yutaka, Hara Kazuo, Mizuno Nobumasa, Kuwahara Takamichi, Okuno Nozomi, Inaba Yoshitaka, Kodaira Takeshi, Abe Tetsuya, Muro Kei, Shimizu Masahito, Niwa Yasumasa (2019). Prognostic Significance of Sarcopenia in Patients with Unresectable Advanced Esophageal Cancer. Journal of Clinical Medicine.

[24] Buzby GP, Williford WO, Peterson OL, Crosby LO, Page CP, Reinhardt GF (1988). A randomized clinical trial of total parenteral nutrition in malnourished surgical patients: the rationale and impact of previous clinical trials and pilot study on protocol design. Am J Clin Nutr.

[25] Mitsiopoulos N, Baumgartner RN, Heymsfield SB, Lyons W, Gallagher D, Ross R (1998). Cadaver validation of skeletal muscle measurement by magnetic resonance imaging and computerized tomography. J Appl Physiol.

[26] Shen W, Punyanitya M, Wang Z, Gallagher D, St-Onge M-P, Albu J (2004). Total body skeletal muscle and adipose tissue volumes: estimation from a single abdominal cross-sectional image. J Appl Physiol.

[27] Palie O, Michel P, Ménard J-F, Rousseau C, Rio E, Bridji B (2013). The predictive value of treatment response using FDG PET performed on day 21 of chemoradiotherapy in patients with oesophageal squamous cell carcinoma. A prospective, multicentre study (RTEP3). Eur J Nucl Med Mol Imaging.

[28] Cosquéric G, Sebag A, Ducolombier C, Thomas C, Piette F, Weill-Engerer S (2006). Sarcopenia is predictive of nosocomial infection in care of the elderly. Br J Nutr.

[29] McMillan DC (2009). Systemic inflammation, nutritional status and survival in patients with cancer. Curr Opin Clin Nutr Metab Care.

[30] Prado CMM, Lima ISF, Baracos VE, Bies RR, McCargar LJ, Reiman T (2011). An exploratory study of body composition as a determinant of epirubicin pharmacokinetics and toxicity. Cancer Chemother Pharmacol.

[31] Awad S, Tan BH, Cui H, Bhalla A, Fearon KCH, Parsons SL, Catton JA, Lobo DN (2012). Marked changes in body composition following neoadjuvant chemotherapy for oesophagogastric cancer. Clinical Nutrition (Edinburgh, Scotland).

